# Integrated transcriptomic and proteomic analysis reveals potential targets for heart regeneration

**DOI:** 10.17305/bjbms.2022.7770

**Published:** 2023-01-06

**Authors:** Liu Liu, Tongtong Yang, Qiqi Jiang, Jiateng Sun, Lingfeng Gu, Sibo Wang, Yafei Li, Bingrui Chen, Di Zhao, Rui Sun, Qiming Wang, Hao Wang, Liansheng Wang

**Affiliations:** 1 Department of Cardiology, The First Affiliated Hospital of Nanjing Medical University, Nanjing, China

**Keywords:** Proteomic, transcriptomic, heart regeneration, myocardial infarction (MI)

## Abstract

Research on the regenerative capacity of the neonatal heart could open new avenues for the treatment of myocardial infarction (MI). However, the mechanism of cardiac regeneration remains unclear. In the present study, we constructed a mouse model of heart regeneration and then performed transcriptomic and proteomic analyses on them. Gene ontology (GO) enrichment analysis, Kyoto Encyclopedia of Genes and Genomes (KEGG) pathway enrichment analysis, and Gene Set Enrichment Analysis (GSEA) of differentially expressed genes (DEGs) were conducted. Western blot (WB) and qPCR analyses were used to validate the hub genes expression. As a result, gene expression at the mRNA level and protein level is not the same. We identified 3186 DEGs and 42 differentially expressed proteins (DEPs). Through functional analysis of DEGs and DEPs, we speculate that biological processes, such as ubiquitination, cell cycle, and oxygen metabolism, are involved in heart regeneration. Integrated transcriptomic and proteomic analysis identified 16 hub genes and Ankrd1, Gpx3, and Trim72 were screened out as potential regulators of cardiac regeneration through further expression verification. In conclusion, we combined transcriptomic and proteomic analyses to characterize the molecular features during heart regeneration in neonatal mice. Finally, Ankrd1, Gpx3, and Trim72 were identified as potential targets for heart regeneration therapy.

## Introduction

As the most drastic manifestation of ischemic heart disease, myocardial infarction (MI) has long been a fatal threat to human health [1]. All existing clinical treatments aim to restore blood supply to the infarcted area, but they cannot save the necrotic myocardium in the infarction zone. Therefore, promoting the endogenous proliferation of cardiomyocytes is considered to be an effective complement to existing treatments [2, 3]. Mice within seven days after birth can initiate cardiomyocyte proliferation and fully regenerate hearts after an MI. However, this regenerative ability vanishes on the seventh day after birth [4]. Exploring the changes in the expression of genes and their encoded proteins during this process can help us unearth the key regulators of heart regeneration [5, 6].

Previous descriptions of the molecular profile of myocardial regeneration models have been limited to the level of the transcriptome [7]. An increasing number of studies have shown that post-translational modifications (PTMs) play important roles in the process of heart regeneration [8]. Therefore, research on the mRNA level alone may not fully reveal the factors that regulate heart regeneration, and elucidating the changes in protein levels will help clarify the mechanism of heart regeneration more deeply [9]. Additionally, compared with mRNA molecules, the key proteins that regulate the regeneration process have more clinical prospects [10, 11].

Given the above consideration, we constructed a heart regeneration model by performing MI surgery on one-day-old mice and then analyzed the transcriptome and proteome at six days post MI (6 dpi). We found that gene expression at the transcript level and the protein level was not completely synchronized. Verification of the expression of key genes, such as Ankrd1, Gpx3, and Trim72, by qPCR and WB analyses indicated that some biological processes (BPs) that regulate translation are involved in heart regeneration. These findings allow us to comprehensively evaluate the connections and differences between mRNA expression and protein expression and how they affect heart regeneration.

## Materials and methods

**Figure 1.**
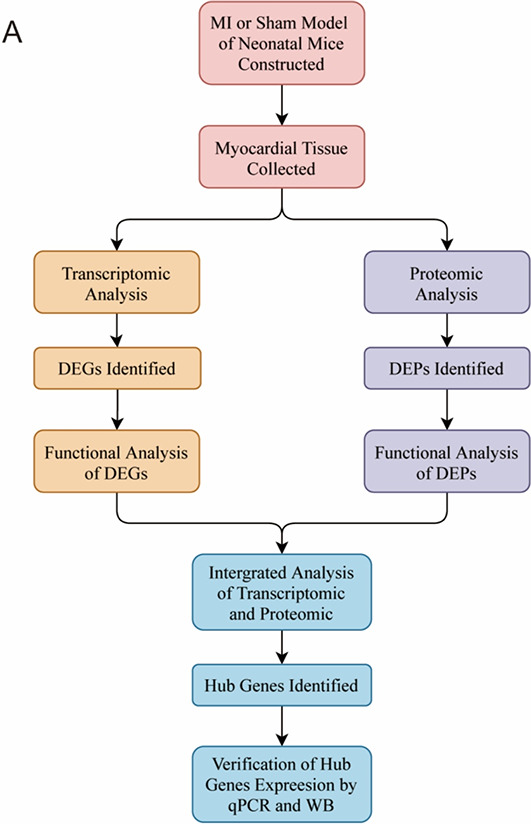
**Workflow chart of the experiments performed in this study.** DEGs: Differentially expressed genes; DEPs: Differentially expressed proteins; WB: Western blot.

A schematic diagram of the experimental workflow is shown in Figure 1.

### MI model of neonatal mice

MI surgery or sham surgery was performed as described previously [12, 13] on one-day-old mice. At 6 dpi, the myocardial tissue from the infarct border zone was collected and subjected to the following experiments.

### Library construction and mRNA sequence analysis

After assessing the purity and integrity of total RNA, we filtered mRNA from total RNA using poly-T oligo-attached magnetic beads. The sequencing library was constructed using NEBNext^®^ UltraTM RNA Library Prep Kit for Illumina^®^ (NEB, USA). After the synthesis of double strands of complementary DNA (cDNA), an AMPure XP system (Beckman Coulter, Beverly, MA, USA) was used to select cDNA fragments from 250 to 300 bp in length. The cDNA fragments were incubated with USER Enzyme (NEB, USA) at 37 ^∘^C for 15 min and then at 95 ^∘^C for 5 min. PCR was performed with Phusion High-Fidelity DNA Polymerase, universal PCR primers, and Index (X) Primer. The PCR products were purified, and library quality was assessed on an Agilent Bioanalyzer 2100.

After using a TruSeq PE Cluster Kit v3-cBot-HS (Illumina) to generate clusters, the constructed library was sequenced on an Illumina Hiseq platform and 150 bp paired-end reads were obtained. The raw data (raw reads) in fastq format were processed through Perl scripts to obtain clean data and then HISAT2 v2.0 was used to map the clean reads to the reference genome. Finally, the fragments per kilobase per million mapped reads (FPKM) value of each gene was calculated with featureCounts v1.5.0-p3 for further analysis.

### Protein preparation and mass spectrometry analysis

The 6 dpi or sham samples were homogenized in SDT buffer (4% SDS, 100 mM Tris–HCl, pH 7.6). Then, the homogenate was centrifuged at 14000× *g* for 40 min, and the supernatant was filtered with 0.22 m filters. A BCA Protein Assay Kit (P0012, Beyotime) was used to quantify the filtrate. After being mixed with 6× loading buffer, proteins for each sample were separated on a 12.5% SDS-PAGE gel. Afterward, 200 µg of protein was reduced with 50 mM DTT for 30 min at 56 ^∘^C, and impurities were removed using UA buffer (8 M urea, 150 mM Tris–HCl pH 8.5) by repeated ultrafiltration (Sartorius, 30 kD). To block reduced cysteine residues, 100 µl of iodoacetamide (IAA;100 mM IAA in UA buffer) was added, and the samples were stored in the dark for 30 min. After being washed three times with 100 µl of UA buffer and twice with 100 µl of 25 mM NH4HCO3 buffer, the proteins were digested with 4 µg of trypsin (Promega) in 40 µl of 25 mM NH4HCO3 buffer overnight at 37 ^∘^C and the peptides were collected as a filtrate the next day.

First, the samples were analyzed on a nanoElute (Bruker, Bremen, Germany). All peptides were separated on a 25 cm × 75 µm analytical column with 1.6 µm C18 beads and a packed emitter tip (IonOpticks, Australia). An integrated column oven (Sonation GmbH, Germany) was used to maintain the column temperature at 50 ^∘^C. The column was equilibrated using four column volumes and then each sample was loaded in 100% buffer A (99.9% Milli-Q water, 0.1% formic acid [FA]). All samples were separated at 300 nl/min using a linear gradient with an increase from 2% to 25% buffer B (99.9% acetonitrile [CAN], 0.1% FA) over 90 min, an increase to 37% buffer B (10 min), an increase to 80% buffer B (10 min) and a hold for 10 min. The total separation time was 120 min.

Then, the samples were analyzed on a timsTOF Pro (Bruker, Bremen, Germany) operated in parallel accumulation–serial fragmentation (PASEF) mode. The settings of the mass spectrometer were as follows: mass range 100 to 1700 m/z, capillary voltage 1500 V, ramp time 100 ms, lock duty cycle to 100%, dry temp 180 ^∘^C, dry gas 3 l/min, 1/K0, start 0.6 Vs/cm^2^ and end 1.6 Vs/cm^2^. The PASEF settings were as follows: 10 MS/MS scans (total cycle time 1.16 s), scheduling target intensity 20,000, intensity threshold 2500, collision-induced dissociation (CID) collision energy 42 eV, charge range 0–5, and active exclusion for 0.4 min.

### Label-free proteomics

MaxQuant software version 1.6.14.0 was used to analyze the MS data. The data were searched against the Uniprot_MusMusculus_17027_20200226 database, which was downloaded at http://www.uniprot.org. The initial search was conducted with a precursor mass window of 6 ppm. The search followed an enzymatic cleavage rule of trypsin/P; two maximal missed cleavage sites and a mass tolerance of 20 ppm for fragment ions were allowed; carbamidomethylation of cysteines was defined as a fixed modification, and protein N-terminal acetylation and methionine oxidation were defined as variable modifications. The false discovery rates (FDRs) for peptide and protein identification were both set to 0.01. Based on the normalized spectral protein intensity (LFQ intensity), protein abundances were calculated. Finally, proteins with fold change values >2 or <0.5 and *p*-values (Student’s t test) <0.05 were identified as differentially expressed proteins (DEPs).

### Bioinformatics analysis of DEGs and DEPs

The DESeq2 R package was used to perform differential expression analysis to identify differentially expressed genes (DEGs). The *P* value was adjusted using Benjamini and Hochberg’s approach to control the FDR. Genes with an adjusted *P* value <0.05 were considered DEGs. Gene Ontology (GO) enrichment analysis, Kyoto Encyclopedia of Genes and Genomes (KEGG) pathway enrichment analysis, and Gene Set Enrichment Analysis (GSEA) of DEGs were all implemented by the clusterProfiler R package, and a cutoff of an adjusted *P* value <0.05 [14].

NCBI-BLAST+ software was used to align protein sequences to the reference database and only the sequences in the top 10 and with an E-value ≤ 1e-3 were retained. The GO terms of the sequences with the top bit-scores were selected and annotated with the Blast2GO Command Line. To improve the accuracy of the annotations, InterProScan was used to search the European Bioinformatics Institute (EBI) database by motif and then add the functional motif information to the proteins. Finally, ANNEX was used to further improve the annotations and connections between GO terms. GO terms enrichment was assessed using Fisher’s exact test by measuring the numbers of DEPs and total proteins correlated with the GO terms.

The GOplot and ggplot2 R packages were used to visualize the GO and KEGG analysis results. Heatmaps were drawn with the pheatmap R package, and a Venn diagram was drawn with the VennDiagram R package.

### Quantitative real-time polymerase chain reaction analysis

Total RNA was extracted with TRIzol reagent (Thermo Fisher Scientific) from the 6 dpi or sham myocardial tissue mentioned above according to the manufacturer’s protocols. The cDNA was synthesized using HiScript^®^ III RT SuperMix for qPCR (R323-01, Vazyme, Nanjing, China). Real-time PCR was performed on an Eppendorf Mastercycler realplex with ChamQ SYBR qPCR Master Mix (High ROX Premixed) (Q341-02, Vazyme, Nanjing, China). All sequences of the primers used in this study are listed in Table S1.

### Western blot analysis

The 6 dpi or sham myocardial tissue was lysed with lysis buffer containing 0.1% protease inhibitor, 1% phosphatase inhibitor, and 0.5% phenylmethylsulfonylfluoride (GeneChem, Shanghai, China). After 20 g of protein from each sample was separated on 10% SDS-PAGE gels (EpiZyme Scientific, Shanghai, China) and the separated proteins were transferred onto polyvinylidene fluoride membranes (Roche Applied Science, Mannheim, Germany), 5% BSA (Solarbio Science & Technology Co., Ltd, Beijing, China) was used to block the membranes. Then, the blocked strips were incubated with primary antibodies overnight at 4 ^∘^C. The primary antibodies used were as follows: anti-Gpx3 (ab256470, 1:1000, Abcam), anti-Ankrd1 (11427-1-AP, 1:4000, Proteintech), anti-Trim72 (22151-1-AP, 1:4000, Proteintech), anti-GAPDH (AP0063, 1:5000, Bioworld), and anti-Tubulin (AP0064, 1:5000, Bioworld). After the membranes were cultured with an HRP-linked anti-rabbit IgG Antibody (1:3000, Cell Signaling Technology, Inc.) at room temperature for 2 h, the proteins on the membranes were visualized with enhanced chemiluminescence (ECL) reagents (FDbio, Hangzhou, China) on an iBright FL1000 Imaging System (Invitrogen, Thermo Fisher Scientific, Waltham, MA, USA). The band intensity was quantified by ImageJ software.

### Ethical statement

All animal experiments met the requirements of the Guide for the Use and Care of Laboratory Animals and were approved by the Animal Care and Use Committee of Nanjing Medical University. Institute of Cancer Research mice on the first day after birth were obtained from the animal center of Nanjing Medical University and fed in a specific pathogen-free animal house.

### Statistical analysis

All the statistical data were analyzed with GraphPad (La Jolla, CA, USA) Prism 8.0 software and were presented as the mean±SEM. To compare mean values between the two groups, unpaired Student’s t tests were performed when the data were shown to be normally distributed. Differences were considered statistically significant if *P* ≤ 0.05.

### Data availability

The data that support the findings of this study are available from the corresponding author on request.

## Results

### Overall statistics of the sequencing data

**Figure S1.**
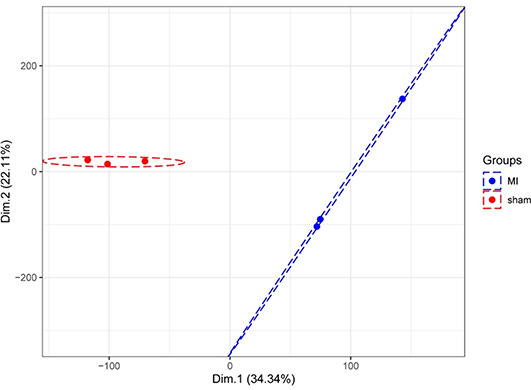
PCA plot of transcriptomic analysis.

We constructed a total of six cDNA libraries, three of which were extracted from myocardial tissue in the MI group and others were extracted from myocardial tissue in the sham group. The overall error rate of each sequencing group was less than 0.2%, and the proportion of clean reads in each sequencing group exceeded 96%. The unique mapping rate ranged between 86.95% and 88.30% when the clean reads were mapped to the reference genome. These findings showed that the quality of the sequencing data was sufficient. We detected 29,241 genes after quantifying the sequenced data. The principal component analysis (PCA) plot is present in Figure S1.

### Identification of DEGs

**Figure 2.**
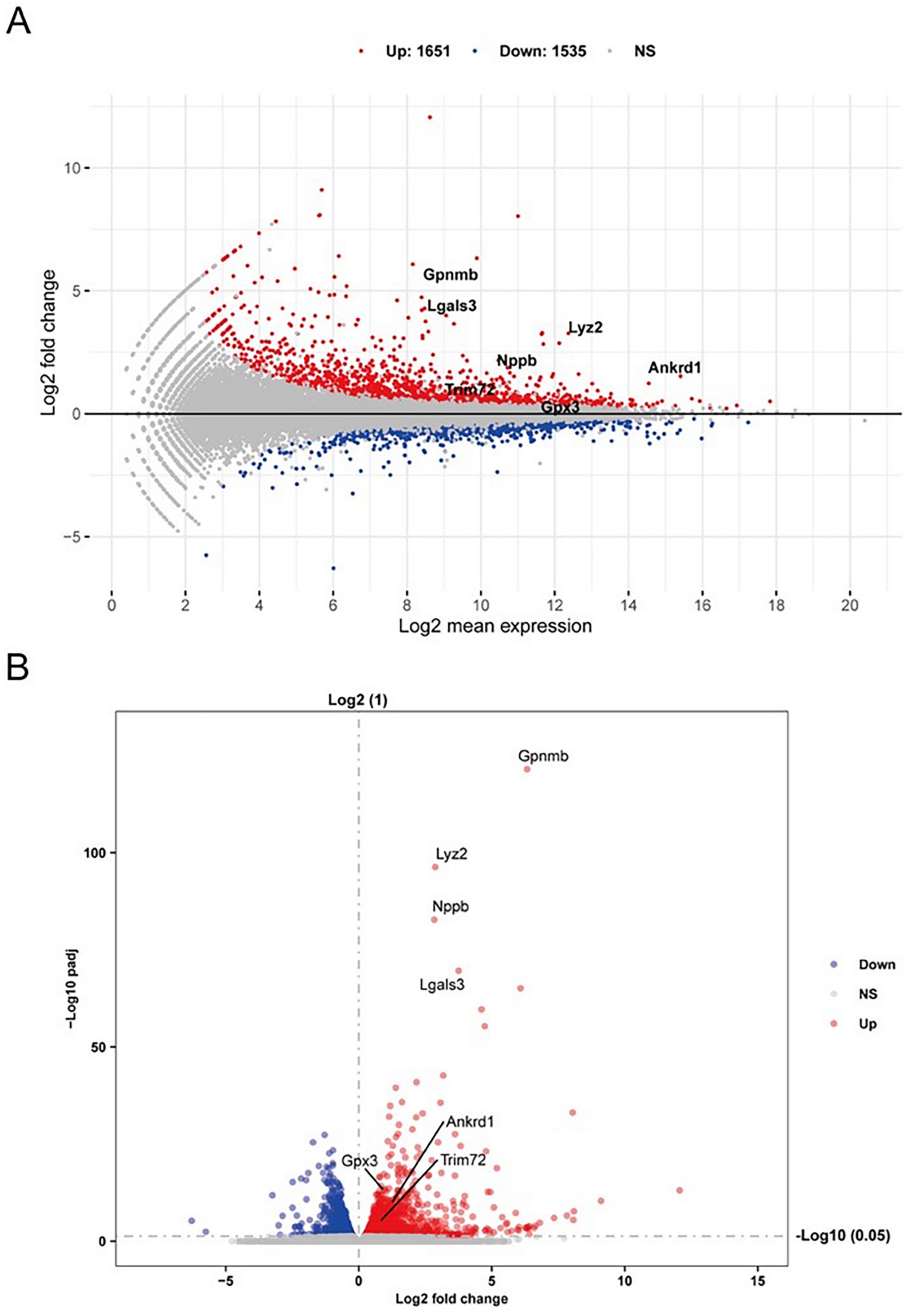
**Expression features of DEGs.** (A) MA plot of DEGs. (B) Volcano plot of DEGs. Red dots represent upregulated genes, and blue dots represent downregulated genes. DEGs: Differentially expressed genes.

**Figure S2.**
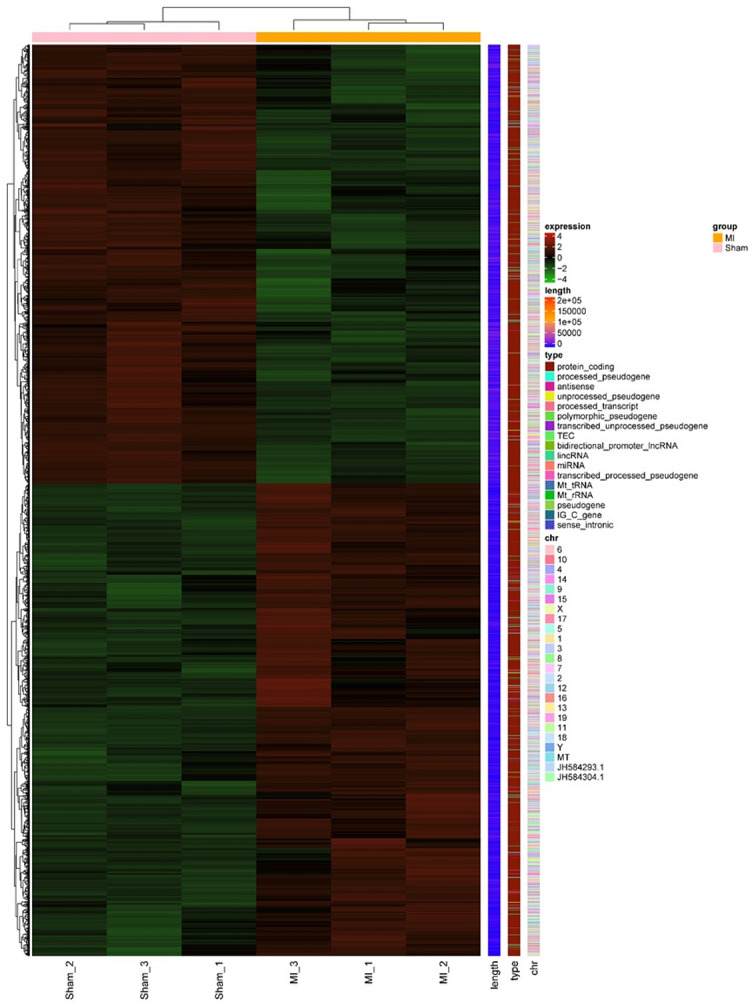
**The heatmap of differentially expressed genes.** Dark red represents upregulated genes and the dark green represents downregulated genes.

After filtering the original sequencing data, we identified 3186 DEGs (1651 upregulated and 1535 downregulated) between the 6 dpi group and the sham group with a standard adjusted *P* value <0.05 using DESeq2 R package. The variance in gene expression is shown using MA plot, volcano plot, and heatmap in Figure 2A and 2B and Figure S2.

### Functional enrichment analysis of DEGs

**Figure 3.**
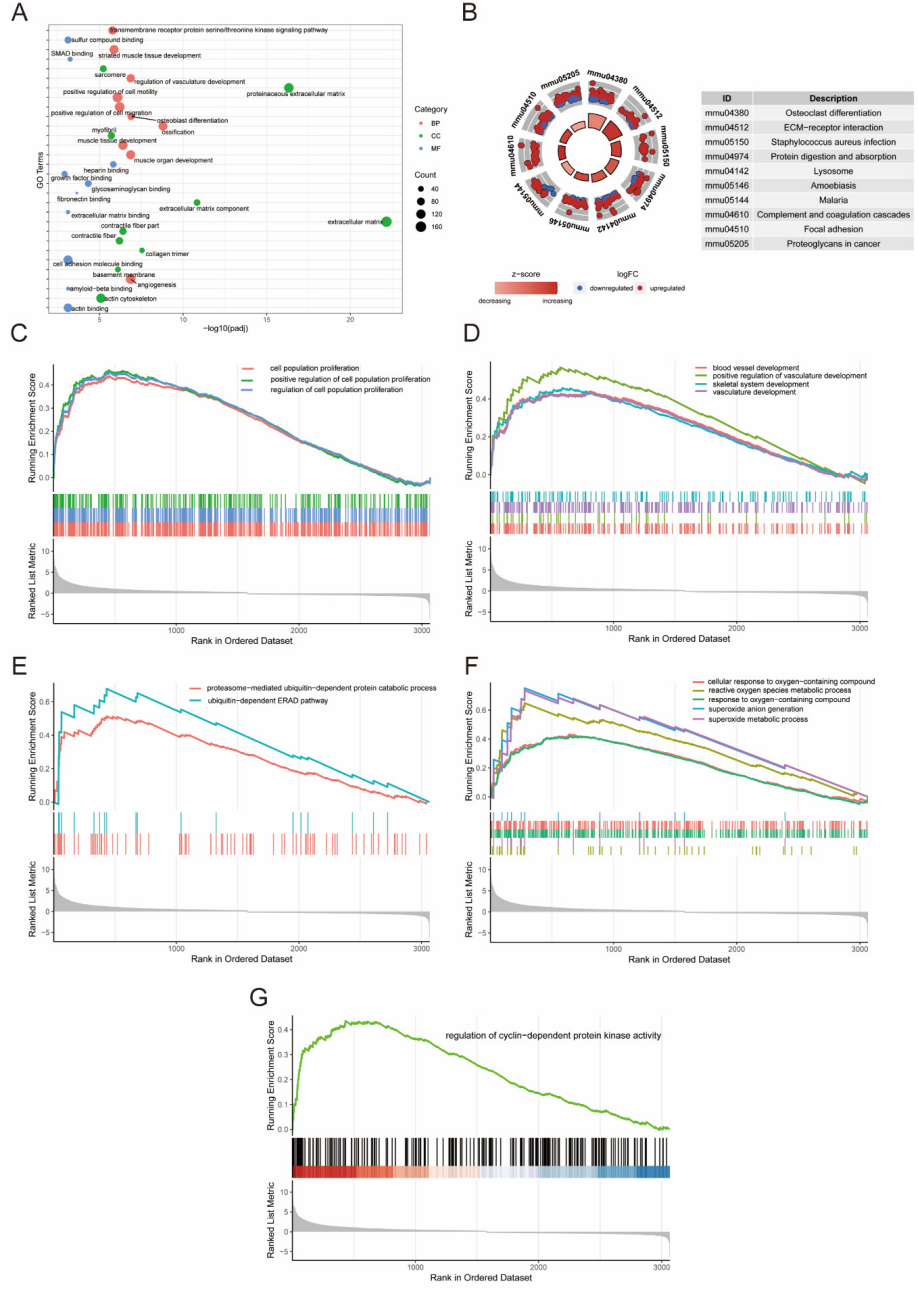
**Functional profiling of DEGs.** (A) Bubble plot of the top 30 enriched GO terms of DEGs. (B) Top 10 enriched KEGG pathways of DEGs. (C)–(G) Results of GSEA of DEGs. DEGs: Differentially expressed genes; GO: Gene ontology; KEGG: Kyoto Encyclopedia of Genes and Genomes.

All 3186 DEGs were subjected to GO enrichment analysis, and the top 10 enriched terms in molecular function (MF), cellular component (CC), and BP categories are shown in Figure 3A. Angiogenesis, muscle organ development, regulation of vasculature development, muscle tissue development, and striated muscle tissue development were highly enriched, which indicated the repair process after heart damage was activated. KEGG pathway enrichment analysis revealed that the DEGs were enriched in pathways, such as ECM-receptor interaction and protein digestion and absorption (Figure 3B). Next, performed GSEA according to the GO database to further identify the biological functions associated with the regeneration process. The results showed that the processes of cell proliferation, oxygen metabolism, ubiquitination, cell cycle, and angiogenesis were enriched (Figure 3C–3G).

### Identification of DEPs

**Figure 4.**
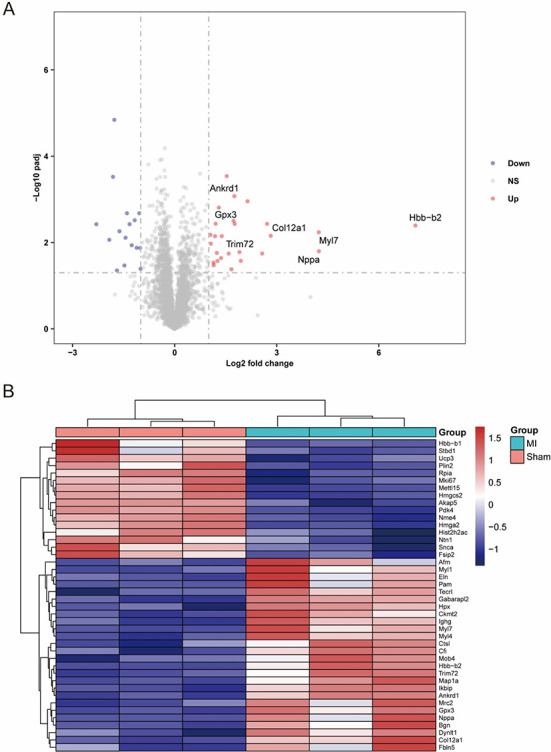
**Expression features of DEPs.** (A) Volcano plot of DEPs. Red dots represent upregulated proteins, and blue dots represent downregulated proteins. (B) Heatmap of DEPs. Red represents upregulated proteins, and blue represents downregulated proteins. DEP: Differentially expressed proteins.

**Figure S3.**
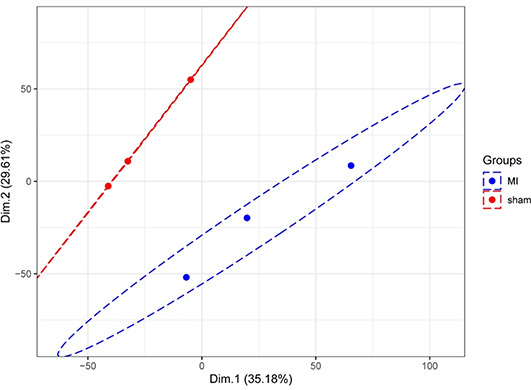
PCA plot of proteomic analysis.

To explore the mechanism of heart regeneration at the protein level, we depicted protein profiling of the MI model and sham model by label-free proteomics analysis. After performing a database search, we identified a total of 39,029 peptides and obtained 4462 protein groups using an FDR ≤ 0.05 as the screening standard for peptides and proteins. The PCA plot of DEPs is shown in Figure S3. Ultimately, 42 proteins (26 upregulated and 16 downregulated) were identified as DEPs for further analysis. The volcano plot and heatmap in Figure 4A and 4B show changes at the protein level.

### Functional enrichment analysis of the DEPs

**Figure 5.**
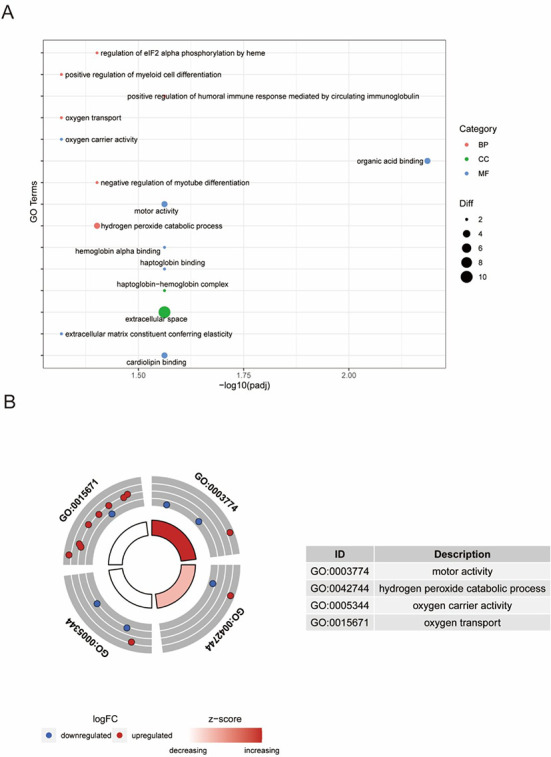
**Functional profiling of DEPs.** (A) Bubble plot of the top 15 enriched GO terms of DEPs. (B) Pathways related to oxygen metabolism were enriched. DEP: Differentially expressed proteins; GO: Gene ontology.

GO enrichment analysis showed that 15 GO terms were significantly enriched for the filtered DEPs. The DEPs were mainly enriched in the hydrogen peroxide catabolic process, oxygen transport, oxygen carrier activity, organic acid binding, and motor activity terms, which indicated that these processes might regulate heart regeneration. The results are shown in Figure 5A and 5B.

### Integrated transcriptomic and proteomic analysis

**Figure 6.**
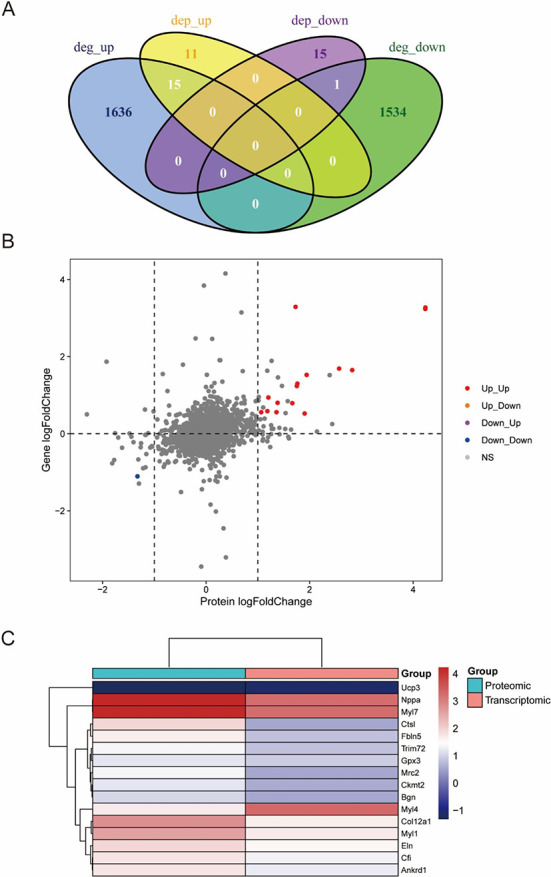
**Comparative and integrative analysis of DEGs and DEPs.** (A) Venn diagram showing the overlap of DEGs and DEPs. (B) Correlation of gene expression at the mRNA level and protein level. The red and blue dots indicate the same trends in expression. (C) The expression of hub genes at the mRNA level and protein level is visualized in a heatmap. DEG: Differentially expressed genes; DEP: Differentially expressed proteins.

Transcriptomic and proteomic analyses provide ways to study gene expression at both the mRNA level and the protein level. Thus, we combined the transcriptomic and proteomic data for further analysis to identify hub genes that might regulate heart regeneration. The correlation plot of the transcriptomic and proteomic data shows the differences in the expression of genes at the mRNA level and the protein level (Figure 6A). As the Venn plot shows in Figure 6B, among 3186 DEGs and 42 DEPs identified in the transcriptome and proteome, only 16 genes had statistical significance at both the mRNA level and the protein level. Of those 16 genes, 15 genes were upregulated and 1 gene was downregulated. The changes in the expression of these genes showed the same trends at the mRNA level and the corresponding protein level. There was no situation in which gene expression was opposite at the two levels. The 16 hub genes were Nppa, Myl7, Col12a1, Myl1, Eln, Ctsl, Cfi, Ankrd1, Myl4, Fbln5, Trim72, Mrc2, Gpx3, Ckmt2, Bgn, and Ucp3. The heatmap in Figure 6C shows the differences in the expression of 16 genes at the two levels.

### Verification of hub gene expression

**Figure 7.**
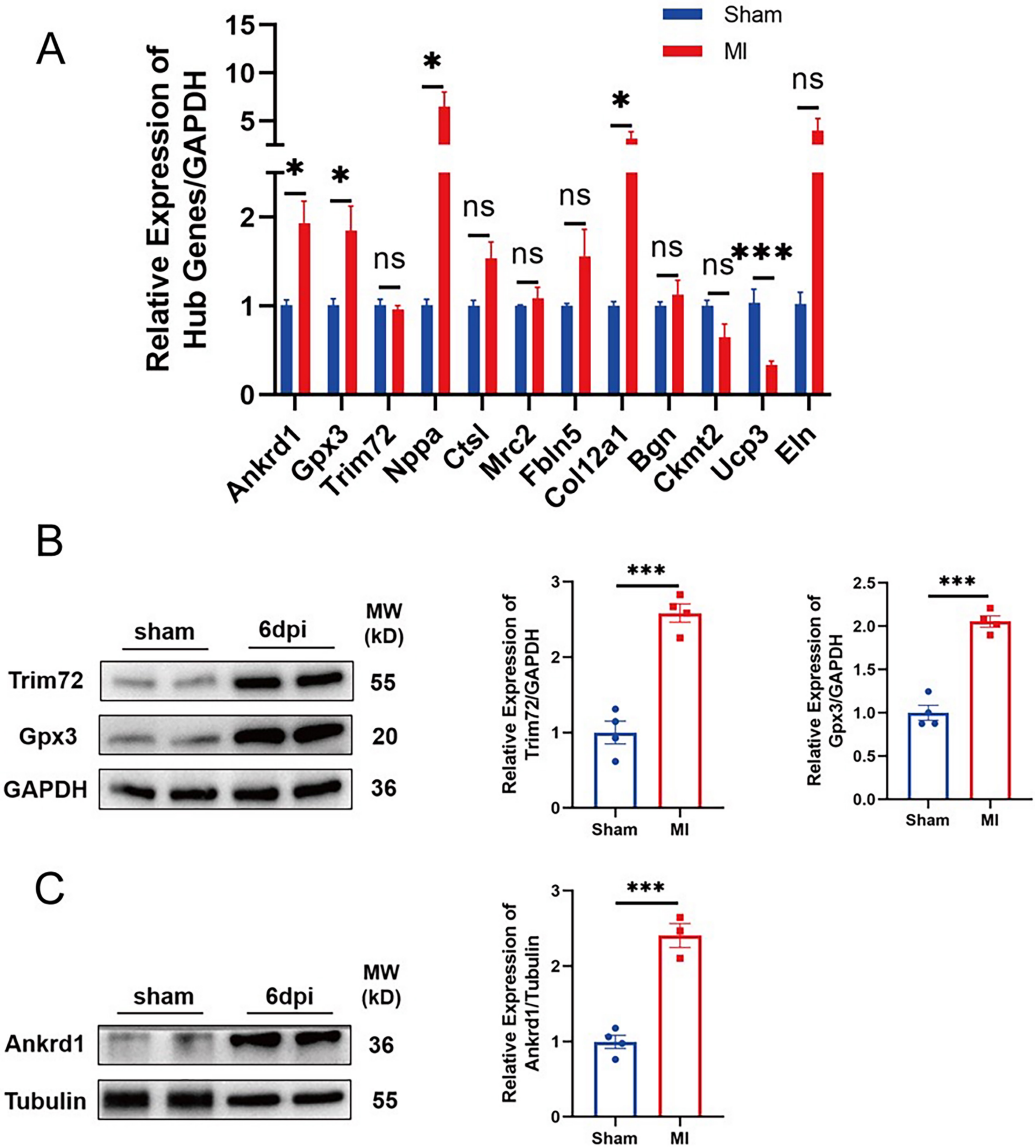
**Verification of hub gene expression.** (A) Differences in the expression of hub genes between the two groups was determined by qPCR (MI group, *n* = 6; sham group, *n* = 4). (B) and (C) WB analysis of Ankrd1, Gpx3, and Trim72 expression in the two groups (MI group, *n* = 4; sham group, *n* = 4). *p < 0.05, ** *p* < 0.01, ***p < 0.001. WB: Western blot; MI: Myocardial infarction.

To further confirm the accuracy of the omics results, we first performed qPCR to detect the expression of the hub genes at the mRNA level between the 6 dpi group and the sham group. The results showed that Ankrd1, Gpx3, Nppa, and Col12a1 were upregulated, while Ucp3 was downregulated (Figure 7A). Subsequently, we carefully explored the relationships between key genes and a series of BPs related to heart regeneration, such as cell proliferation. Ankrd1, Gpx3, and Trim72 were identified as potential regulators in heart regeneration and the WB results showed that the three proteins were upregulated in the 6d pi group (Figure 7B–7C).

## Discussion

Endogenous myocardial regeneration therapy is considered to be a promising and powerful supplement for the treatment of MI [15, 16]. Neonatal mice can completely regenerate their hearts from damage within seven days after birth, and thus are excellent models for studying heart regeneration therapy [17–19]. Although a previous experiment attempted to elucidate the molecular mechanism of the regeneration process, it was limited to the mRNA level [7]. Therefore, in the present study, we performed transcriptomic and proteomic analyses together to reveal the expression profiles of genes and proteins in heart regeneration models to identify potential pivotal regulators. A total of 3186 significant DEGs (1651 upregulated and 1535 downregulated) were identified, and 42 significant DEPs (26 upregulated and 16 downregulated) were identified. These results showed that the expression trends of the transcriptomic and proteomic profiles were not the same. Protein expression did not completely correspond to gene expression at the mRNA level. Accordingly, studying the consistent or inconsistent associations between gene and protein expression could help reveal key regulators that regulate heart regeneration. In addition, we could verify the results of a previous transcriptomics study [7].

The results of GO enrichment analysis and GSEA showed that DEGs were highly enriched in BPs related to cardiomyocyte development and proliferation, such as angiogenesis, muscle organ development, muscle tissue development, striated muscle tissue development, and cell population proliferation. GO enrichment analysis of DEPs yielded similar results: BPs related to oxygen metabolism were enriched. These results implied possible regulatory mechanisms for heart regeneration. Then, we conducted an integrated transcriptomic and proteomic analysis to screen out key genes. Sixteen proteins matched their corresponding genes. Through qPCR and WB verification experiments, we identified Ankrd1, Gpx3, and Trim72 as hub genes.

Changes in cardiac energy metabolism from neonatal mice to adult mice have been confirmed to affect the regeneration of cardiomyocytes. Neonatal mice produce energy primarily through glycolysis, while adult mice produce energy through fatty acid oxidation [20]. This shift in metabolism leads to the accumulation of reactive oxygen species (ROS) and further leads to cell cycle arrest through the DNA damage response. Notably, timely elimination of ROS can extend the time window for cardiomyocyte regeneration [21]. One study has confirmed that exposing adult mice to a hypoxic environment for a week after MI can improve the function of the left ventricle of the heart by reducing mitochondrial metabolism, DNA damage, and ROS production [22]. The above findings show that the level of oxygen metabolism in cardiomyocytes may be a novel target for the promotion of cardiomyocyte proliferation. The results of our study showed that oxygen metabolism-related processes were enriched. Thus, it is meaningful to determine a target for intervention in this process. Glutathione peroxidase 3 (Gpx3) is a crucial antioxidant defense molecule in cardiomyocytes, which could neutralize ROS and then maintain the homeostasis of cardiomyocytes, protecting them from excessive accumulation of ROS [23]. Loss of Gpx3 is related to the occurrence of cardiovascular diseases. In patients with atrial fibrillation, a decline of Gpx3 was associated with an increased risk of cardiovascular events [24]. Similarly, Gpx3 knockdown could cause damage to cardiomyocytes through autophagy and apoptosis due to ROS accumulation [25]. These studies indicated that Gpx3 can play a protective role in cardiomyocytes and it is likely a powerful target for the promotion of cardiomyocyte regeneration.

Heart regeneration can be initiated by the re-entry of cell cycle-arrested cardiomyocytes into the cell cycle [26]. The results of the GSEA of the DEGs showed that regulation of cyclin-dependent protein kinase activity was enriched. Therefore, we sought to identify key genes associated with cyclins and cyclin kinases. In adult zebrafish, which can regenerate their hearts throughout their lives, Ankrd1 is upregulated following cardiac injury in the border zone of cardiomyocytes [27]. In addition, Y-box Protein 1 (YB1) has been confirmed to promote the expression of cyclin A2/B1 in the nucleus and further promote the proliferation of cardiomyocytes [28, 29]. Ankrd1 is a potential upstream regulator of YB1, and the nuclear localization signal (NLS) sequence in Ankrd1 regulates protein nuclear translocation from the cytoplasm to the nucleus [30, 31]. Taking these findings into consideration, we believe that Ankrd1 is likely an effective target for intervention in the process of heart regeneration.

The regulatory potential of protein PTMs in heart regeneration has received increasing attention. Differences between proteomic and transcriptomic results can reflect the effects of PTMs. Thus, comparative proteomic and transcriptomic analysis is an excellent way to study PTMs. Ubiquitination, one of these modifications, can regulate cell growth, proliferation, and differentiation by mediating protein expression, activation, and degradation [32]. Ubiquitination has been reported to regulate cyclins and cyclin-dependent kinases to activate the cell cycle, thus it might play a vital role in myocardial regeneration [33]. Ubiquitination can promote cardiomyocyte proliferation by upregulating the ubiquitination of proteins that inhibit cardiomyocyte regeneration and reducing the ubiquitination of proteins that promote cardiomyocyte regeneration [34, 35]. Trim72 is a striated muscle-specific tripartite motif (TRIM) family protein that plays a dual role in the occurrence and development of cardiovascular diseases [36]. Many studies have demonstrated the positive role of Trim72 in cell proliferation; for example, it can target a series of downstream factors, such as P53, CAV1, TGF-β, and NDRG-2, to mediate cell proliferation [37–40]. However, the role of Trim72 in the proliferation of cardiomyocytes has not yet been reported. The increase in Trim72 at the mRNA level in the 6 dpi group compared with the sham group was not significant, but the increase at the protein level was significant. Therefore, it is likely that Trim72 may be regulated by PTM. Based on the above findings, the mechanism of Trim72 upregulation and Trim72 function during cardiac regeneration deserve further study.

There are still some other aspects of this research that need to be further explored. First, we need to clarify the correlation between the expression of key genes and the time window for cardiomyocyte regeneration. Then the expression of key genes needs to be artificially regulated to verify their functions in heart regeneration.

To the best of our knowledge, our study is the first to combine transcriptomic and proteomic analyses to fully characterize the transcriptome and proteome of neonatal mice heart regeneration. Our multi-omics data underline multiple mechanisms that may regulate cardiac regeneration and identify Gpx3, Ankrd1, and Trim72 as new potential targets for the treatment of MI.

## Conclusion

In conclusion, we combined transcriptomic and proteomic analyses to characterize the molecular features during heart regeneration in neonatal mice. Ankrd1, Gpx3, and Trim72 were identified as potential targets for heart regeneration therapy.

**Conflicts of interest:** The authors declare no conflicts of interest.

**Funding:** This work was supported by grants from the Natural Science Youth Foundation of Jiangsu Province of China (BK20191068), the National Natural Science Foundation of China (No. 82070367), and a Project Funded by the Priority Academic Program Development of Jiangsu Higher Education Institutions (PAPD, No. KYZZ15_0263).

## Figures and Tables

**Table S1:** The sequences of primers used in qPCR

**Primer(mouse)**	**Sequence (5’to3’)**
GAPDH forward primer	AGGTCGGTGTGAACGGATTTG
GAPDH reverse primer	TGTAGACCATGTAGTTGAGGTCA
Ankrd1 forward primer	GCTGGTAACAGGCAAAAAGAAC
Ankrd1 reverse primer	CCTCTCGCAGTTTCTCGCT
Gpx3 forward primer	CCTTTTAAGCAGTATGCAGGCA
Gpx3 reverse primer	CAAGCCAAATGGCCCAAGTT
Trim72 forward primer	GGAAGATGCGGATGTTCCTGG
Trim72 reverse primer	GCTCTAGGTAAGAGTTCAGGCT
Nppa forward primer	ACCCTGGGCTTCTTCCTCGTCTT
Nppa reverse primer	GCGGCCCCTGCTTCCTCA
Ucp3 forward primer	GTTTACTGACAACTTCCCCT
Ucp3 reverse primer	CTCCTGAGCCACCATCT
Mrc2 forward primer	TCTCCCGGAACCGACTCTTC
Mrc2 reverse primer	AACTGGTCCCCTAGTGTACGA
Fbln5 forward primer	GGAGGTTACACCTGCTCCTG
Fbln5 reverse primer	CACAGAGCTGCTGGCAGTAA
Col12a1 forward primer	GTCATACACTCTCAAATTCCTCAC
Col12a1 reverse primer	GACACTCCATACACCATCACG
Bgn forward primer	ACTTCTGTCCTATGGGCTTCGG
Bgn reverse primer	GCTTCTTCATCTGGCTATGTTCCTC
Eln forward primer	GGCCATTCCTGGTGGAGTTCC
Eln reverse primer	AACTGGCTTAAGAGGTTTGCCTCC
Ctsl forward primer	CCCTATGAAGCGAAGGACGG
Ctsl reverse primer	CTGGAGAGACGGATGGCTTG
Ckmt2 forward primer	ACCCCGGCCACCCCTTCATTA
Ckmt2 reverse primer	GCCAGCCAGATCGCCCTTCAG
